# A novel cancer vaccine strategy with combined IL-18 and HSV-TK gene therapy driven by the hTERT promoter in a murine colorectal cancer model

**DOI:** 10.3892/ijo.2014.2557

**Published:** 2014-07-22

**Authors:** KOSUKE HIGASHI, SHOICHI HAZAMA, ATSUHIRO ARAKI, KIYOSHI YOSHIMURA, NORIO IIZUKA, SHIGEFUMI YOSHINO, TAKAFUMI NOMA, MASAKI OKA

**Affiliations:** 1 Department of Digestive Surgery and Surgical Oncology, Yamaguchi University Graduate School of Medicine, Ube, Yamaguchi 755-8505, Japan; 2 Department of Molecular Biology, Institute of Health Biosciences, The University of Tokushima Graduate School, Tokushima 770-8503, Japan

**Keywords:** interleukin-18, HSV-TK, hTERT promoter, colorectal cancer, minimal residual tumor

## Abstract

A therapeutic vaccine against minimal residual cancer cells is needed for the treatment of patients with colorectal cancer. Several gene therapy studies have revealed that the combination of a suicide gene and cytokine gene might induce effective antitumor immunity. In this study, we constructed an interleukin (IL)-18 and herpes simplex virus-thymidine kinase (HSV-TK) expression vector driven by the human telomerase reverse transcriptase (hTERT) promoter to study the efficacy of combination gene therapy with IL-18 and the HSV-TK suicide gene. Low immunogenic colon 26 cells were used for transfection and inoculation into syngeneic BALB/c mice. Large established tumors of colon 26 transfectants expressing IL-18 and HSV-TK driven by the hTERT promoter were completely eradicated after GCV administration in syngeneic BALB/c mice. Immunohistochemical analysis at the tumor rejection sites revealed enormous infiltrations of CD8^+^ T lymphocytes as well as CD4^+^ T lymphocytes and CD11b^+^ monocytes. Moreover, established distant tumors were completely eradicated by vaccination with the IL-18 and HSV-TK transfectants in combination with GCV. These data suggest that the IL-18 and suicide gene therapy can elicit antitumor specific immunity. In conclusion, gene therapy with IL-18 and HSV-TK plasmid vector driven by the hTERT promoter may be useful for cancer vaccination.

## Introduction

Colorectal cancer (CRC) is the second leading cause of cancer-related death in industrialized countries (1). In the last decade, the combined regimens of multiple anticancer drugs have markedly improved the survival of patients with CRC at stages III and IV (2). However, ~30% of patients with CRC at stage III experience disease recurrence, even if a curative resection of the primary tumor has been performed (3). Hence, a novel strategy against minimal residual tumors is strongly desired.

Interleukin-18 (IL-18) is a proinflammatory cytokine that is mainly secreted from antigen-presenting cells (APCs). IL-18 induces interferon (IFN)-γ secretion from a number of cell types in the immune system (4–6), stimulates T-cell proliferation, and augments NK cell lytic activity (7). Gene therapy with IL-18 activates the cytotoxicity of CD8^+^ T cells (8–10) and also activates CD4^+^ T cells (9) and NK cells (10).

The transfer of suicide genes, such as herpes simplex virus-thymidine kinase (HSV-TK), into a variety of tumor cells exerts antitumor efficacy (11). The antitumor activity is the result of the activation of ganciclovir (GCV) to its cytotoxic triphosphate derivative that induces apoptosis in target cells (12), which results in tumor antigen exposure to immune cells that leads to antitumor specific immunity (13). However, suicide gene therapy is thought to be insufficient, and the combination of suicide gene therapy and cytokine gene therapy may be preferable. The efficacy of the antitumor effects may be improved by the combination of HSV-TK with cytokine genes, such as IL-2 (14,15), IL-12 (16,17), or granulocyte-macrophage colony-stimulating factor (14). However, there have been no previous studies to examine the IL-18 and HSV-TK suicide gene combination. We have previously demonstrated that a mature-IL-18 expression vector with the Igκ leader sequence is useful for construction of clones that secrete a large amount of bioactive IL-18 and that IL-18-secreting tumor cells can elicit a specific antitumor immune response via the infiltration of CD4^+^ T cells and CD8^+^T cells (9). The TK/GCV system results in tumor mass reduction, and local secretion of IL-18 may maintain the Th1 immunity and tumor-specific cytotoxic T lymphocytes (CTLs) induced by the APCs that process tumor antigens from the apoptotic tumor cells killed by the TK/GCV system.

On the other hand, IL-18 has strong toxicity, such as fulminant hepatitis induced by intravenous administration of IL-18 (18). The localization of cytokines at the site of tumors may decrease the adverse effects; similarly, the HSV-TK suicide gene should be expressed only on tumor cells. Therefore, research in gene therapy has been focused on the development of targeting strategies. The discovery of telomerase, its high prevalence in tumor tissues, and its transcriptional regulation via the human telomerase reverse transcriptase (hTERT) promoter has extended the applicability of gene therapy vectors (19). Hence, we have investigated hTERT promoter activities (20) and have used the hTERT promoter to achieve tumor-specific gene expression of a target transgene.

In the present study, we constructed a plasmid vector co-expressing HSV-TK and IL-18 driven by the hTERT promoter. We confirmed the tumor-specific promoter activity of the hTERT promoter and the antitumor effect mediated by the compound expression of HSV-TK and IL-18. We demonstrated the therapeutic usefulness of this vector against distant metastatic tumors by vaccinating with low immunogenic colon 26 cells transfected with our novel vector.

## Materials and methods

### RT-PCR and primers

RNA isolation and reverse transcriptase- polymerase chain reaction (RT-PCR) were performed as previously described with some modifications (21). Briefly, cells (5×10^6^) were solubilized in 1 ml of TRIzol reagent (Life Technologies, Grand Island, NY, USA), and total cellular RNA was isolated according to the manufacturer’s instructions. Total RNA (1 μg) was added to 19 μl of RT-mixture (Takara, Ohtsu, Japan). After mixing, the samples were incubated at 30°C for 10 min, 55°C for 30 min, 95°C for 5 min, and 4°C for ≥5 min. PCR-mixture (80 μl) (Takara) containing 100 nM primers was added to the RT products. PCR cycling parameters were as follows for hTERT promoter, IL-18 and HSV-TK expansion, respectively; hTERT cDNA: denaturation at 94°C for 30 sec, annealing at 68°C for 30 sec, and extension at 72°C for 2 min utilized 35 cycles. IL-18 cDNA: denaturation at 94°C for 1 min, annealing at 60°C for 1 min, and extension at 72°C for 1.5 min utilized 28 cycles. HSV-TK cDNA: denaturation at 94°C for 30 sec, annealing at 64°C for 30 sec, and extension at 72°C for 2 min for 33 cycles. These primer sequences were as follows: hTERT-F primer (5′-GGC GGC ATT AAT GGC CCC TCC CTC GGG TTA CCC-3′) and R primer (5′-TTA TTA GCT AGC CGC GGG GGT GGC CGG GGC CAG-3′); IL-18-F′ primer (5′-GAG AAG CTT AAC TTT GGC CGA CTT CAC TGA-3′) and R′ primer (5′-CGC CTC GAG CTA ACT TTG ATG TAA GTT AGT-3′); and HSV-TK-F′ primer (5′-GAC TCT AGA CGT ATG GCT TCG TAC CCC TGC CAT-3′) and R′ primer (5′-AGT GTC GAC GTT TCA GTT AGC CTC CCC CAT CTC-3′). The expected sizes of the PCR products were 444 bp for hTERT promoter, 492 bp for IL-18 cDNA, and 1,137 bp for HSV-TK cDNA, respectively. These PCR products were subjected to 1% agarose gel electrophoresis and visualized by staining with ethidium bromide.

### Construction of plasmid vector

**Figure 1:**
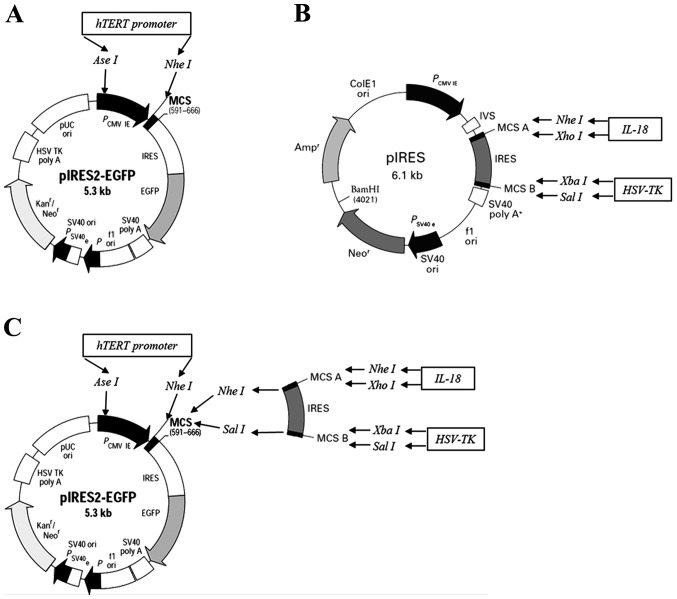
Construction of hTERT-IL18 vector, hTERT-TK vector and hTERT-IL18-TK vector. Details are described in Materials and methods. IL-18, interleukin- 18; HSV-TK, herpes simplex virus-thymidine kinase; MCS, multiple cloning site.

The full-length cDNA of the hTERT promoter for which promoter activity had been confirmed by chloramphenicol acetyltransferase (CAT) assay was kindly provided by Professor T. Noma (Yamaguchi University School of Medicine, Japan). CAT assays (unpublished data) revealed that the region from -444 to 0 of the hTERT promoter was required for maximal promoter activity. This hTERT promoter region was isolated by PCR with the CAT vector as a template. The PCR products of the hTERT promoter were cloned into the *Ase*I/*Nhe*I site of the pIRES2-EGFP vector (Clontech, Palto Alto, CA, USA) to create the hTERT-EGFP vector (Fig. 1A and Table I).

Full length IL-18 is processed into its mature active form by caspase-I; thus, it is difficult to engineer secreted active IL-18. Hence, we previously constructed a novel plasmid vector in which the murine mature-IL-18 coding region was driven by the Igκ leader sequence to stimulate the secretion of active IL-18 (9). A mature IL-18 cDNA with Igκ leader sequence was digested by *Nhe*I/*Xho*I, and the fragments were cloned into the *Nhe*I/*Xho*I site of the pIRES vector (Fig. 1B).

pBluescript SK (+) vector including the full-length coding region of HSV-TK was kindly provided by Dr T. Okada (Jichi Medical University, Japan). HSV-TK cDNA was digested with *Xba*I/*Sal*I, and a 1.2-kb insert was cloned into the *Xba*I/*Sal*I site of the pIRES vector (Fig. 1B). IL-18 cDNA, HSV-TK cDNA, or IL-18 and HSV-TK cDNA were digested at the *Nhe*I/*Sal*I site of the pIRES vector and cloned into the hTERT-EGFP vector to create hTERT- IL18, hTERT-TK or hTERT-IL18-TK vector, respectively (Fig. 1C and Table I). The nucleotide sequences were confirmed by sequence analysis with an ABI 1620 Genetic Analyzer (Applied Biosystems, Tokyo, Japan).

### Tumor cells, normal fibroblasts, and transfection

We have established human esophageal cancer cell lines YES-2, YES-3, YES-5, and YES-6 (22) and YPK-1 and YPK-4 (23) in our department. We purchased SNV398, MKN28 and MKN74 from American Type Culture Collection (ATCC, Sumitomo Farma International, Tokyo, Japan). These human cancer cell lines were maintained in Dulbecco’s modified Eagle’s medium (Nissui Pharmaceutical, Tokyo, Japan) supplemented with 10% heat-inactivated fetal calf serum (FCS), 100 U/ml penicillin G, and 100 μg/ml streptomycin. Colon 26, a murine colon adenocarcinoma cell line derived from BALB/c mice (24), and normal human fibroblasts derived from the epidermis of surgical specimens were maintained in RPMI-1640 medium (Life Technologies) supplemented with 10% FCS. Subconfluent cultures in 33-mm 6-well plates were transfected with 2 μg of each plasmid vector by using Lipofectamine 2000 reagent (Life Technologies) according to the manufacturer’s instructions. G418 (100 mg/ml) (Life Technologies) was added to the cells 48 h later. G418-resistant clones were isolated and expanded in culture medium containing 100 mg/ml of G418.

### Analysis of gene expression

The intensity of EGFP expression in fibroblasts and cancer cell lines transfected with pIRES2-EGFP vector and hTERT-EGFP vector was analyzed by fluorescence microscopy and an EPICS flow cytometer (Coulter Electronics, Hialeah, FL, USA).

The levels of IL-18 secreted from each transfectant into the culture supernatant were measured by using an enzyme-linked immunosorbent assay (ELISA) (BioSource, Camarillo, CA, USA) according to the manufacturer’s instructions. Cells (1×10^6^ cells/ml) transfected with pIRES2-EGFP, hTERT-TK, hTERT-IL18, or hTERT-IL18-TK vector were cultured without G418 for 48 h. Culture supernatants were collected by centrifugation for 5 min at 400 g. Each sample was assayed in duplicate. The lower limit of the detection of mature-IL-18 was <1 pg/ml.

To detect the expression of HSV-TK, GCV was added to subconfluent cultures of each clone of colon 26 cells transfected with hTERT-TK vector or TERT-IL18-TK vector and parent colon 26 cells in 33-mm 6-well plates at a concentration ranging from 0 to 2,000 μM (2,000, 1,000, 500, 250, 125, 62.5, 31.25, 15.6 and 7.8 μM). From three to seven days later, the sensitivity of the transfectants or parental cells to GCV was analyzed by fluorescence microscopy.

### Animal experiments

Seven-week-old female BALB/c mice were purchased from Japan SCL (Hamamatsu, Japan). The tumorigenicity of the hTERT-TK-transfected colon 26 clone number 1 (hTERT-TK1) or hTERT-IL18-TK-transfected colon 26 clone number 52 (hTERT-IL18-TK52) or colon 26 parental cells was examined by subcutaneous injections into BALB/c mice. Injection of colon 26 cells was performed with freshly prepared suspensions, and all injections were subcutaneous in the right and/or left lower abdominal quadrant via a 27-gauge needle (25). Tumor volumes were measured in mm^3^ with a vernier caliper and determined according to the following formula: a × b^2^/2, where a is the larger and b the smaller of the two dimensions. All animal experiments were conducted in accordance with the guidelines of the Animal Care and Use Committee of Yamaguchi University School of Medicine.

### Histological evaluation of immune cells infiltrating the tumor tissues

Tumor cell injection sites were dissected, fixed in 10% neutral buffered formalin, and embedded in paraffin. Sections of 4-μm thickness were stained with hematoxylin and eosin. For immunohistochemical staining, tissues were embedded in OCT compound (Ames Division, Miles Laboratories, Elkhart, IN, USA), snap-frozen in liquid nitrogen, and stored at −80°C. Acetone-fixed 6-mm cryostat sections were blocked with goat serum and then immunostained with the optimal dilution of the following rat mAbs: L3/T4 (CD4: Becton-Dickinson, Franklin Lakes, NJ, USA), KT15 (CD8: Serotec, Sapporo, Japan), and M1/70 (CD11b: Becton- Dickinson). The slides were then sequentially incubated with biotinylated goat anti-mouse IgG (Zymed Laboratories, South San Francisco, CA, USA) and ABComplex (Dako, Tokyo, Japan). Each incubation step was ≥30 min and was followed by a 10-min PBS wash. Sections were then incubated with 0.03% H_2_O_2_ and 0.06% 3.3′-diaminobenzidine for 2–5 min, washed in tap water, and counterstained in hematoxylin.

### Statistical analysis

Statistical analysis was performed by one-way analysis of variance (ANOVA) and analysis of covariance. A value of p<0.01 was considered statistically significant. Results are presented as mean ± standard error (SE).

## Results

### Gene expression of EGFP, IL-18, and HSV-TK

**Figure 2:**
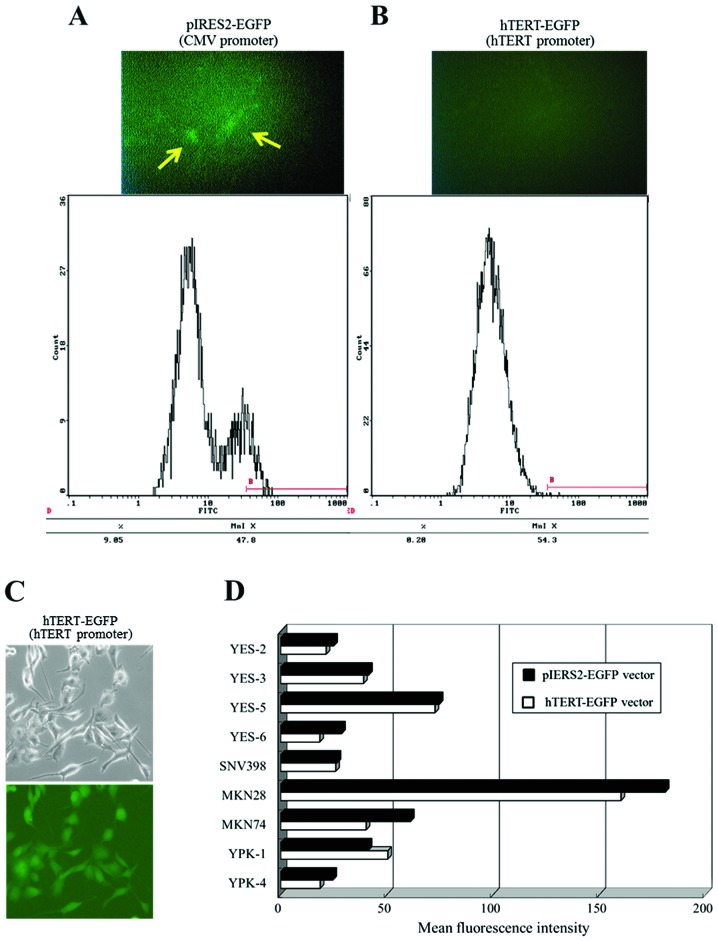
Expression of GFP by each transfectant. (A) The assessment of EGFP expression in pIRES2-EGFP vector-transfected fibroblasts. Upper images show the GFP-positive expression of fibroblasts by fluorescence microscopy. Lower images show the positive expression of GFP by flow cytometry. (B) Upper images show the negative expression of GFP on fibroblasts by fluorescence microscopy. Lower images show the negative expression of GFP by flow cytometry. In normal cell lines, TERT-EGFP-transfected fibroblast clones did not express EGFP. (C) Upper, optical microscopy; lower, fluorescence microscopy. In the cancer cell lines, the GFP expression level of TERT-EGFP-transfected colon 26 cells was high. (D) Mean fluorescence intensity of GFP on various human cancer cell lines transfected with pIRES-EGFP vector and hTERT-EGFP vector was determined by flow cytometry.

GFP expression levels of pIRES2-EGFP- and hTERT-EGFP-transfected fibroblasts are shown in Fig. 2A and B. Although GFP expression was observed in fibroblasts transfected with pIRES2-EGFP vector carrying the CMV promoter (Fig. 2A), no GFP expression was observed in fibroblasts transfected with hTERT-EGFP vector (Fig. 2B). Contrary to fibroblasts, GFP expression levels in hTERT-EGFP-transfected colon 26 clones were clear (Fig. 2C). We compared the GFP expression in pIRES2-EGFP-transfected cells and hTERT EGFP-transfected cells by using various types of cancer cell lines. GFP expression levels in tumors derived from the two types of transfectants were nearly identical (Fig. 2D). The promoter activity of the CMV promoter and hTERT promoter was thought to be identical.

**Figure 3:**
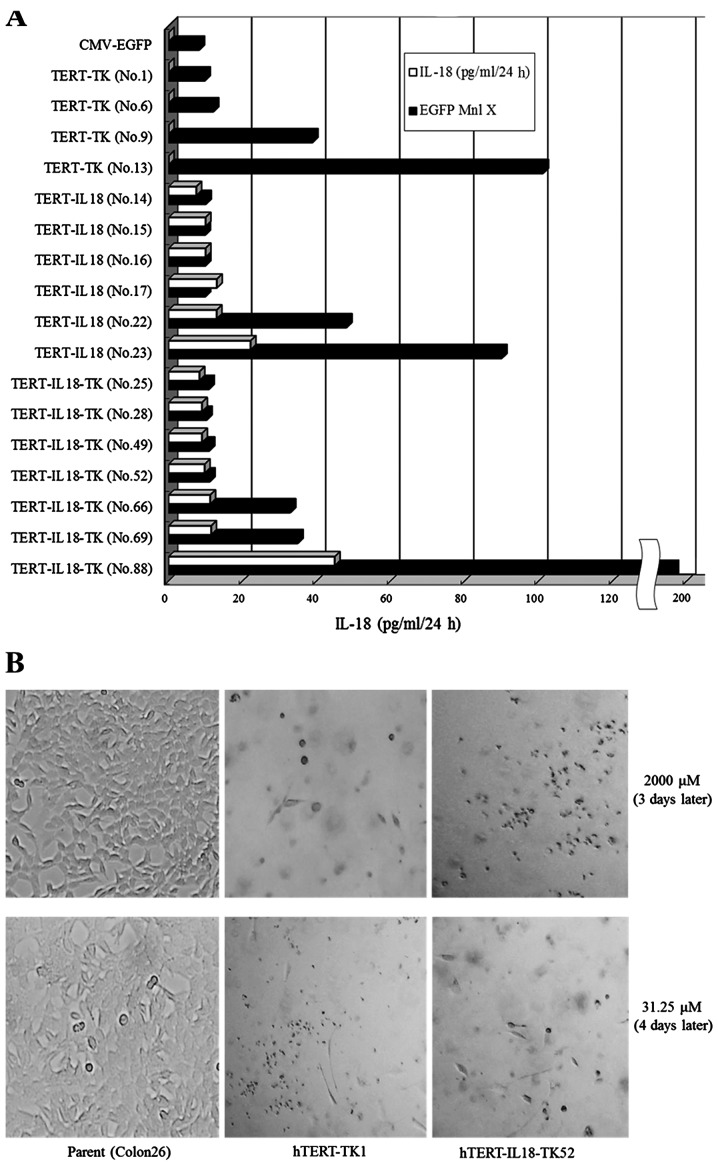
IL-18, GFP, and HSV-TK expression of each colon 26 transfectant. (A) IL-18 levels (pg/ml) in the supernatants from each transfectant (1×10^6^ cell/24 h) and GFP expression in each transfectant. IL-18, interleukin-18; CMV-EGFP, pIRES2-EGFP vector transfectant of colon 26 cells; TERT-TK, TERT-IL18 and TERT-IL18-TK are explained in Table I. (B) Optical microscopy findings for each clone. Details are described in Results.

We constructed hTERT-IL18-TK, hTERT-IL18 and hTERT-TK plasmid vectors driven by hTERT promoter (Fig. 1 and Table I). Colon 26 cells were transfected with the above-mentioned vectors or with pIRES2-EGFP vector driven by the CMV promoter. Independent G-418-resistant transfectants, TERT-TK-transfected colon 26 clones (TERT-TK1 to 13), hTERT-IL18-transfected colon 26 clones (TERT-IL18- 14 to 23), hTERT-IL18-TK-transfected colon 26 clones (hTERT-IL18-TK25 to 88), were isolated and expanded. The expression levels of GFP and secretion of mature IL-18 in each clone are shown in Fig. 3A. We selected clones with moderate gene expression levels, the hTERT-TK1 clone and hTERT-IL18-TK52 clone, for further experiments (Fig. 3A). The expression of GFP and production of IL-18 by the transfectants was stable for >11 months, and production fluctuated <5% in each clone.

The sensitivities to GCV of parental colon 26, hTERT-TK1 clone, and hTERT-IL18-TK52 were examined. Parental colon 26 cells were viable without any morphologic changes ≤4,000 μM of GCV for 7 days (Fig. 3B). On the contrary, hTERT-TK1 as well as hTERT-IL18-TK52 cells died at the concentration of GCV (31.25 μM) after 4 days (Fig. 3B).

### Animal studies

TERT-TK1 (TK1) cells or TERT-IL18-TK52 (IL18-TK52) cells did not alter the growth properties of parental colon 26 cells *in vitro* as assessed by doubling time or morphology (data not shown).

### Study 1: comparison of the antitumor effect of IL-18 alone, HSV-TK with GCV alone, and the combination of IL-18 and HSV-TK with GCV

**Figure 4:**
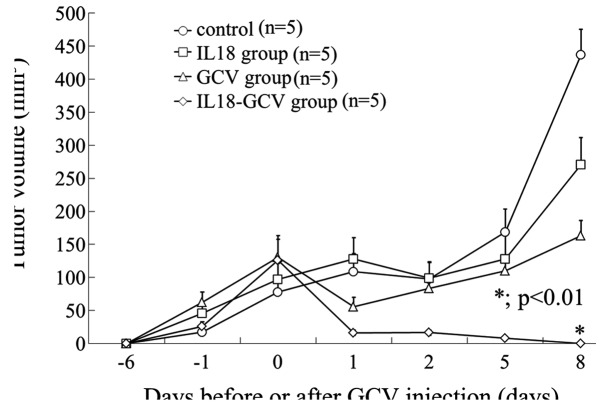
*In vivo* growth of transfected colon 26 cells. The tumorigenicity of hTERT-TK1 and hTERT- IL18-TK52 was examined by subcutaneous injections into BALB/c mice. TK1 and IL18-TK52 without GCV were the control group and IL-18 group, respectively. TK1 and IL18-TK52 with injections of GCV 30 mg/kg were the GCV group and the IL18-GCV group, respectively. The mean tumors of the IL-18-GCV group were eradicated completely (n=5), whereas tumors of the control group (n=5), GCV group (n=5) and IL-18 group (n=5) grew progressively. ^*^p-value of <0.01 for the IL-18-GCV group versus the other three groups.

The tumorigenicity of TK 1 or IL18-TK52 was examined by subcutaneous injections into BALB/c mice. TK1-inoculated mice and IL18-TK52-inoculated mice without GCV injection (control group and IL18 group, respectively) and TK1-inoculated mice and IL18-TK52-inoculated mice with GCV injections (GCV group and IL18-GCV group, respectively) were studied (Fig. 4). Injections of tumor cells were performed with freshly prepared suspensions at a concentration of 2×10^7^ cells/ml. The total number of tumor cells injected per animal was 2×10^6^ cells, which was increased 10-fold as compared to our previous study (9). Intraperitoneal GCV injections of 30 mg/kg were performed on 7 consecutive days from day 7 to 13 after tumor cell inoculation. The established tumors of the IL18-GCV group were eradicated completely (n=5), whereas tumors of the control group (n=5), the GCV group (n=5), and the IL-18 group (n=5) grew progressively (Fig. 4).

### Re-challenge with parental colon 26 cells to reveal acquired immunity

Forty days after the disappearance of the initial IL18-TK52 tumor cells, eight mice were injected with 1×10^6^ parental colon 26 cells in the lower left abdominal flank, resulting in the rejection of tumors in all mice (data not shown).

### Histology at the site of tumor cell injection

**Figure 5:**
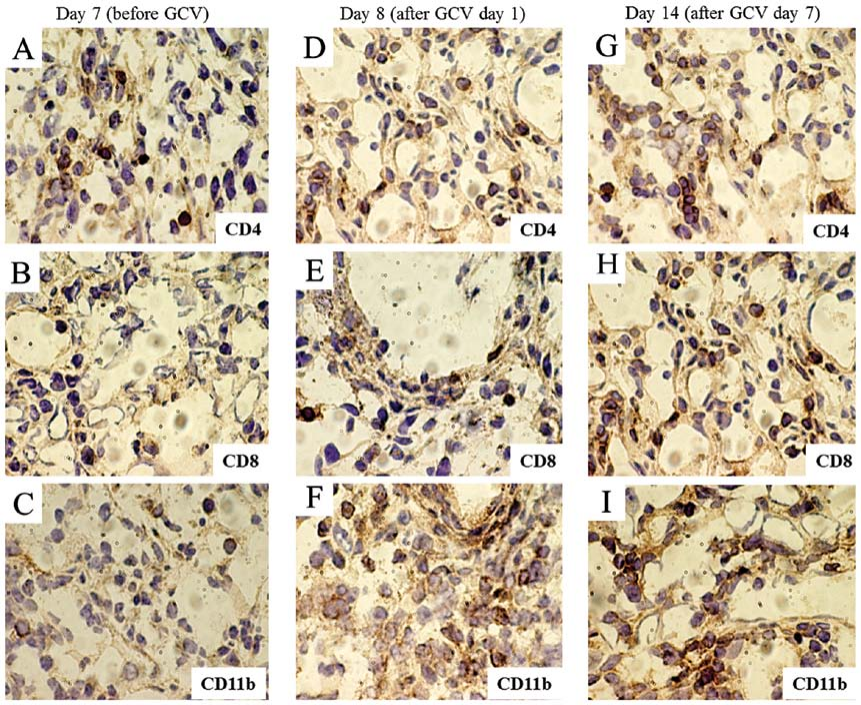
Immunohistochemical findings of the injection sites of IL18-TK52 cells. Immunohistochemical analysis of tumor injection sites in BALB/c mice on days 7, 8 and 14 after subcutaneous injection of IL18-TK52 cells.

To characterize the host cellular responses induced by the combination of IL-18 and suicide gene therapy, immunohistochemical analysis of the injection site was performed on days 7, 8 and 14 following the injection of tumor cells (Fig. 5). On day 7, before GCV injection, moderate infiltration of CD4^+^ lymphocytes (Fig. 5A) was observed at the site of injection of IL18-TK52 cells. This infiltration was not observed in the control group (data not shown). On day 8, one day after GCV injection, moderate infiltration of CD 11b-positive monocytes and CD8^+^ lymphocytes in addition to the CD4^+^ lymphocytes (Fig. 5D–F) was observed. On day 14, 7 days after GCV injection, massive infiltration of CD8^+^ lymphocytes and CD 11b-positive monocytes as well as CD4^+^ lymphocytes was observed (Fig. 5G–I).

### Study 2: immuno-gene therapy using IL-18 and HSV-TK double-transfected tumor cells against an established distant tumor as a model for post-operative gene therapy against minimal residual tumors

**Figure 6:**
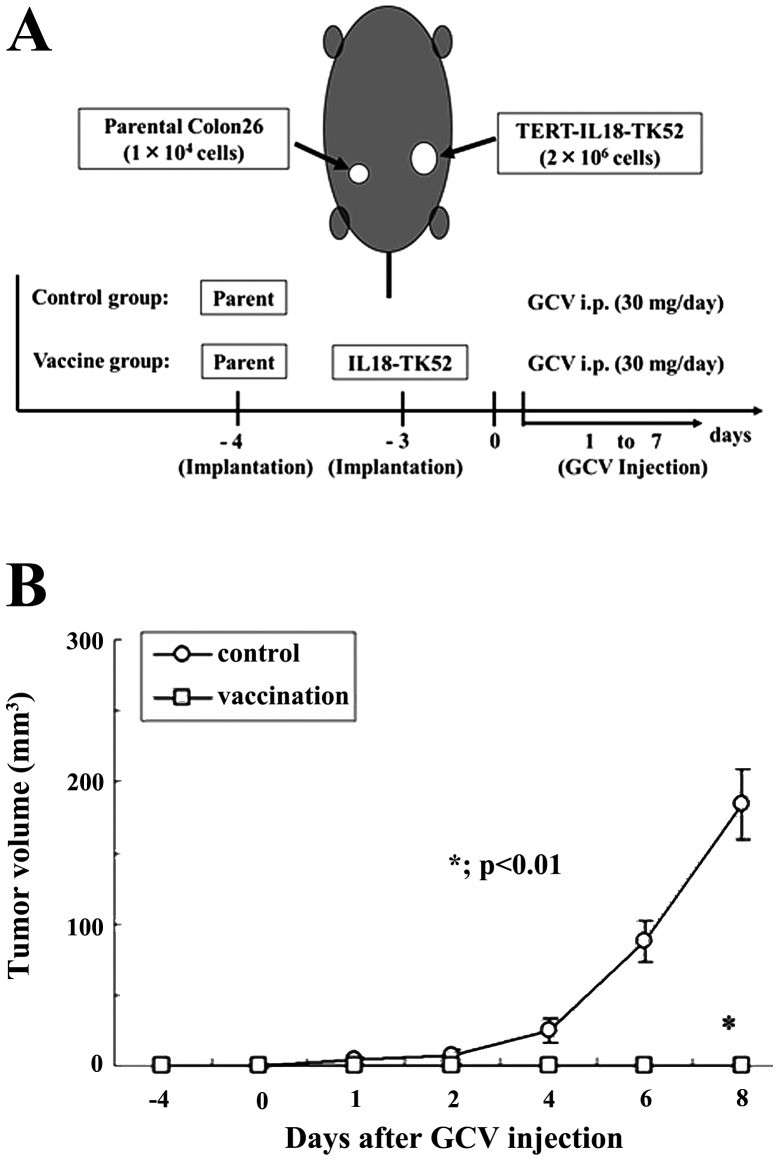
Immuno-gene therapy with IL-18 and HSV-TK double-transfected tumor cells. (A) The scheme of the experimental study of vaccination against minimal residual tumors. GCV, ganciclovir. (B) The mean tumor volumes and standard errors of the control group and vaccination group. In the vaccination group, tumors were eradicated completely (n=5), whereas in the control group (n=5), tumors grew progressively.

As a minimal residual tumor model, 1×10^4^ parental colon 26 cells were inoculated into the right lower abdominal quadrant. The control group received 30 mg/kg of GCV five days after parent cell inoculation on 7 consecutive days. The vaccination group was inoculated with 2×10^6^ IL18-TK52 cells the day after the initial inoculation of parental colon 26 cells in the opposite (left) lower abdominal quadrant. The vaccination group also received 30 mg/kg of GCV, which was the same dosage used in the control group (Fig. 6A).

The tumors in the vaccination group (n=5) were completely eradicated, whereas the tumors in the control group (n=5) grew progressively (p<0.01, Fig. 6B).

## Discussion

In the present study, we evaluated whether gene therapy with both IL-18 and HSV-TK is more effective than gene therapy with IL-18 or HSV-TK alone for the induction of specific anti-tumor immunity to treat distant tumors and suppress primary tumor growth. The topics of our investigation are the specific promoter activity of the hTERT promoter and the synergistic effects of IL-18 and suicide gene therapies.

First, we evaluated hTERT promoter activity. EGFP expression levels of hTERT-EGFP-transfected human tumor cell lines were identical to those of pIRES2-EGFP (bearing CMV promoter)-transfected human tumor cell lines (Fig. 2D). In normal cell lines, TERT-EGFP-transfected fibroblast clones did not express EGFP, and only CMV-EGFP-transfected fibroblast clones expressed EGFP (Fig. 2A and B). Thus, the hTERT promoter was specific enough to be used in targeting transgene expression to tumors.

Second, the strong synergistic effect of the IL-18 and TK/GCV system was demonstrated. IL-18 gene therapy alone was successful with a small-volume inoculation of tumor cells, as described previously (9); but, in the present study, 10-fold the number of tumor cells was inoculated, and tumors grew progressively after IL-18 gene therapy by itself. TK/GCV-suicide gene therapy alone resulted in tumor volume reduction, but the remnant tumor mass grew again. On the other hand, combination gene therapy of the IL-18 and TK/GCV system resulted in the complete rejection of a large tumor mass (Fig. 4).

To clarify the mechanisms of combination therapy, we examined the histological findings of tumor cell injection sites. In the control group, which was identical to mock transfectants, there was very little inflammatory cell infiltration at the injection site, which is consistent with our previous results from mock-transfected colon 26 cells (9). In the IL-18 and TK/GCV combination gene therapy group, infiltration of CD4^+^ T cells at the injection site of IL18-TK52 cells (Fig. 5A) indicated that the local pre-treatment of IL-18 might maintain the T-lymphocyte status around the tumor. There were few CD8^+^ T cells and CD11b^+^ macrophages (Fig. 5B and C). On day 8, one day after GCV administration, a massive infiltration of macrophages was observed (Fig. 5F). On day 14, at the site of the complete rejection of the large tumor mass after GCV administration, there was an enormous infiltration of CD8^+^ T cells as well as CD4^+^ T cells and monocytes (Fig. 5G–I). These findings indicated that GCV administration in the IL18-GCV group induced the following phenomenon: augmentation of immune cell infiltration (9), collapse of tumor mass by apoptosis (12), processing of tumor antigens from the apoptotic tumor cells by recruited APCs that lead to antigen spreading (26), and induction of tumor-specific CTL (7,27). Apoptotic tumor cells induced by the TK/GCV system were also reported to enhance APC functions to release antitumor cytokines such as IL-12 and IL-18 (28). IL-18 secreted by tumor cells may shift CD4^+^ T cells toward the Th1 subset and induce tumor-specific CTL activity (9,28), independently of IL-12 (29). IL-18 may also enhance FasL-mediated cytotoxicity and FasL expression on effector cells that induce apoptosis in Fas-expressing target cells (6). On the other hand, the TK/GCV system would induce downregulation of Bcl-2 and upregulation of caspase-9/-3 in the tumor cells, which increases the sensitivity to apoptosis induced by CTL (30). The IL-18 and TK/GCV dual gene therapy system may have synergistic efficacy by increasing the sensitivity of target cells as well as the tumor-specific CTL activity and the accumulation of immune cells.

The vaccination efficacy of combination gene therapy with both IL-18 and the TK/GCV system indicates rejection of parental cell re-challenge in all mice immunized with IL18-TK52 cells revealing acquired immunity (data not shown). Moreover, established distant tumors were completely eradicated by vaccination with IL18-TK52 cells (Fig. 6). The enormous amount of infiltrated CD8^+^ T cells at the IL18-TK52 rejection site 7 days after GCV administration may support the establishment of tumor-specific acquired immunity. The locally induced tumor-specific CTLs may circulate through the whole body and be preferentially localized at the distant tumor site.

In conclusion, this combination gene therapy strategy is attractive because it harnesses the body’s own defense mechanisms without severe toxicities, and it has the potential to destroy distant tumors via antitumor specific immunity. IL-18 and HSV-TK combination gene therapy with the hTERT promoter may provide a new strategy of cancer gene therapy for the adjuvant therapy after curative resection of primary colorectal cancer.

## Figures and Tables

**Table I:** Plasmid vectors used in this study.

Vector construction	Abbreviations	Promoter	Inserts
pIRES	pIRES	CMV	None
pIRES2-EGFP	pIRES2-EGFP	CMV	EGFP
pIRES2-hTERT-EGFP	hTERT-EGFP	hTERT	EGFP
pIRES2-hTERT-IL-18-EGFP	hTERT-IL18	hTERT	IL-18, EGFP
pIRES2-hTERT-HSV-TK-EGFP	hTERT-TK	hTERT	HSV-TK, EGFP
pIRES2-hTERT-IL-18-HSV-TK-EGFP	hTERT-IL18-TK	hTERT	IL-18, HSV-TK, EGFP

EGFP, enhanced green fluorescent protein; IRES, internal ribosome entry sites; hTERT, the human telomerase reverse transcriptase promoter; TK (HSV-TK), herpes simplex virus-thymidine kinase; IL-18, interleukin-18.
